# Characterization of Pure Ozonides from Ozonolysis of Oleic Acid Methyl Ester; Use of a Protocol for the Analysis of the Corresponding Stable Ozonides from Triolein and Organic Extra Virgin Olive Oil (+OIL^®^)

**DOI:** 10.3390/molecules30030507

**Published:** 2025-01-23

**Authors:** Serena Vella, Marina DellaGreca, Angela Tuzi, Flavio Cermola

**Affiliations:** 1 Erbagil s.r.l., Via L. Settembrini, 13, 82037 Telese Terme, Italy; s.vella@erbagil.com; 2 Dipartimento di Scienze Chimiche, Università di Napoli Federico II, Complesso Universitario di M. Sant’Angelo, Via Cintia, 80126 Napoli, Italy; dellagre@unina.it (M.D.); angela.tuzi@unina.it (A.T.)

**Keywords:** stable ozonides, ozonolysis, ozone, oleic acid methyl ester, triolein, +OIL, Ozoile

## Abstract

As part of the research directed at establishing the composition of the main products present in Ozoile^®^, a study of the ozonolysis of the oleic acid methyl ester as well as triolein was undertaken. Starting from oleic acid methyl ester, all six ozonides were isolated for the first time and fully characterized. Then, we used a protocol based on ozonolysis of triolein and +OIL followed by trans-esterification of the crude reaction mixtures, which led to the same six ozonides. Furthermore, to exclude the formation of any other oxygenated compounds, both during the ozonolysis process and afterward, the reactivity towards ^3^O_2_ and ^1^O_2_ was explored. The ozonolysis of oleic acid methyl ester in a participating solvent (MeOH) was also investigated.

## 1. Introduction

There are many studies conducted on the ozonolysis of olive or vegetable oils aimed at understanding the composition of the ozonides present in the reaction mixtures [1,2,3]. The interest is linked to the pharmacological properties of ozonated vegetable oils which, thanks to their enhanced antimicrobial and anti-inflammatory activity, represent a promising therapeutic possibility for the treatment of skin diseases and topical infections. Research shows that oils such as ozonated olive oil can promote tissue regeneration and counteract various skin pathogens, with clinical applications ranging from the treatment of inflammatory skin diseases to the management of chronic wounds and ulcers [4,5,6,7]. Methyl oleate has also been the subject of studies in ozonolysis reactions, as it is widely present in the form of triglyceride in extra virgin olive oils. The six ozonides obtained were separated and characterized spectroscopically as *cis*/*trans* pairs of each diastereoisomer but were never isolated and characterized separately [8,9,10].

Here, we examine Ozoile (stable Ozonides), a pool of oxygen-rich molecules of a lipid nature obtained through green technology according to a patented process (EP 3900821- Erbagil s.r.l.), through the reaction of ozone with the olefin bonds of the fatty acids of the organic extra virgin olive oil (+OIL). Ozoile delivered by various pharmaceutical forms for topical use (oleolite, cream, emulsion, hydrogel) has been proposed for the treatment of various skin and mucous membrane pathologies, highlighting tissue regenerating and repairing activity. Indeed, Ozoile modulates cellular redox balances through stable ozonides, inducing moderate transient oxidative stress that stimulates the body to produce antioxidants, triggering cascade processes of tissue repair [11,12,13,14,15].

Since determining the composition of Ozoile is very complicated due to the complexity of the biological extra virgin olive oil (+OIL) matrix subjected to ozonolysis, we developed a protocol based on the study of the ozonolysis of oleic acid methyl ester (**1**) as well as triolein (**2**), the latter chosen as the model of the main component of +OIL. The course of the ozonolysis of **2** was compared by performing a methanol trans-esterification of the crude ozonized mixture. The same method was applied to the crude Ozoile. All diastereoisomers of each ozonide have been isolated for the first time and fully characterized.

Furthermore, to obtain information on the relative amount of the two reaction intermediates, the ozonolysis of **1** was performed using methanol as the participating solvent. Initially, the reactivity of **1** and +OIL towards ^3^O_2_ was investigated, as well as of **1** towards ^1^O_2_, to verify the absence of additional oxygenated compounds formed during and after the ozonolysis process.

## 2. Results and Discussion

Ozonolysis is a well-known 1,3-dipolar cycloaddition of ozone (O_3_) to C-C double bonds that leads to a primary ozonide, which in turn rearranges into the final ozonide, a 1,2,4-trioxolane (Criegee reaction).

**Figure 1:**
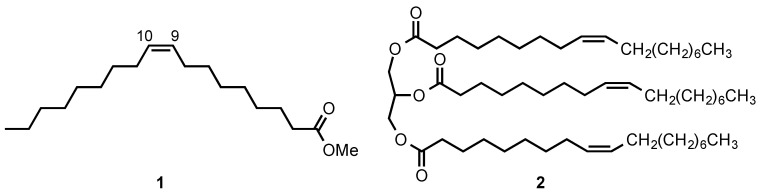
Structures of methyl oleate (**1**) and triolein (**2**).

Here, the results obtained by carrying out the ozonolysis of **1** and **2** (Figure 1), as well as of +OIL, are reported.

We initially investigated the reactivity of **1** and +OIL towards triplet oxygen (^3^O_2_), which is always present under the ozonization procedure used. 

First, compound **1** was treated with ^3^O_2_ in two different ways. An aliquot of **1** was dissolved in dichloromethane (DCM) and oxygen was slowly bubbled for 4 h through the solution kept at 0 °C. A second aliquot was left at room temperature in contact with air for ten days. Then, the mixtures were analyzed by NMR spectroscopy. The ^1^H NMR spectra of both the crude reaction mixtures showed the presence of only starting material **1**. The same trend was found starting from +OIL, which was treated with the same procedures.

**Scheme 1:**
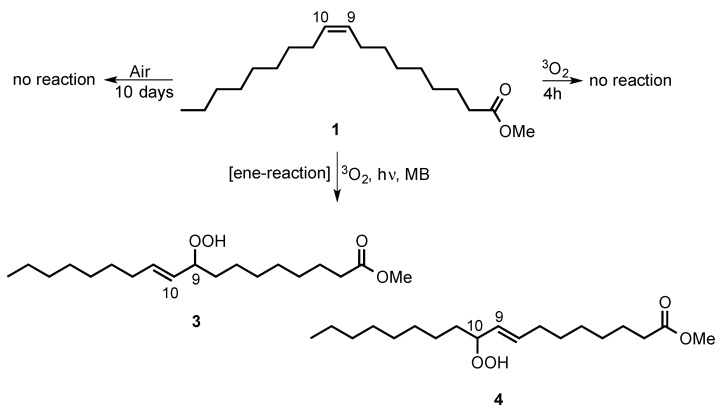
Reactivity of methyl oleate (**1**) towards singlet and triplet oxygen.

Successively, a solution of **1** in dry DCM (0.05 M) was photooxygenated at −20 °C following a reported procedure [16]. After 5 h, the ^1^H NMR spectrum showed the presence of a small amount of unsaturated compounds deriving from the ene-reaction, in addition to the starting material **1** present as the main compound. A silica gel chromatography afforded the starting compound **1** and the hydroperoxides **3** and **4**, whose structures were assigned by comparing their spectral data with those reported in the literature (Scheme 1) [17]. No compounds deriving from a [2+2]-cycloaddition of singlet oxygen [18] to **1** were found, not even in traces.

**Scheme 2:**
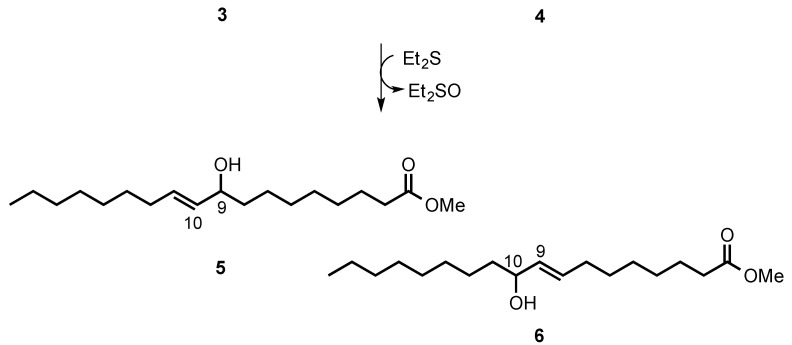
Reduction of hydroperoxides **3** and **4** by diethyl sulfide.

Reduction of a mixture of **3** and **4** by Et_2_S afforded the corresponding allylic alcohols **5** and **6** (Scheme 2).

The allylic alcohols **5** and **6** were recognized through a comparison of their NMR data with those reported in the literature [17,19].

Next, the first ozonolysis experiment was carried out using a 0.25:1 molar ratio of O_3_ to **1**. The NMR analysis of the crude reaction mixture highlighted the presence of signals attributable to the expected ozonides, in addition to the starting material that was the main component. Here, the competition of the ene-reaction was excluded because the corresponding products were not detected in the ^1^H NMR spectrum.

Despite the presence of a sensitizer such as chlorophyll in olive oil extracts, this result was the expected one as the ozonolysis process is very rapid and occurs in the absence of light. Additionally, a check carried out after leaving the ozonated mixture in the presence of sunlight and oxygen for some days showed the absence of allyl hydroperoxides, even in trace amounts. This control and the previous one relating to the lack of products deriving from oxidation with ^3^O_2_ were necessary, as the patented ozonolysis process of +Oil is also carried out in ozone deficiency.

**Scheme 3:**
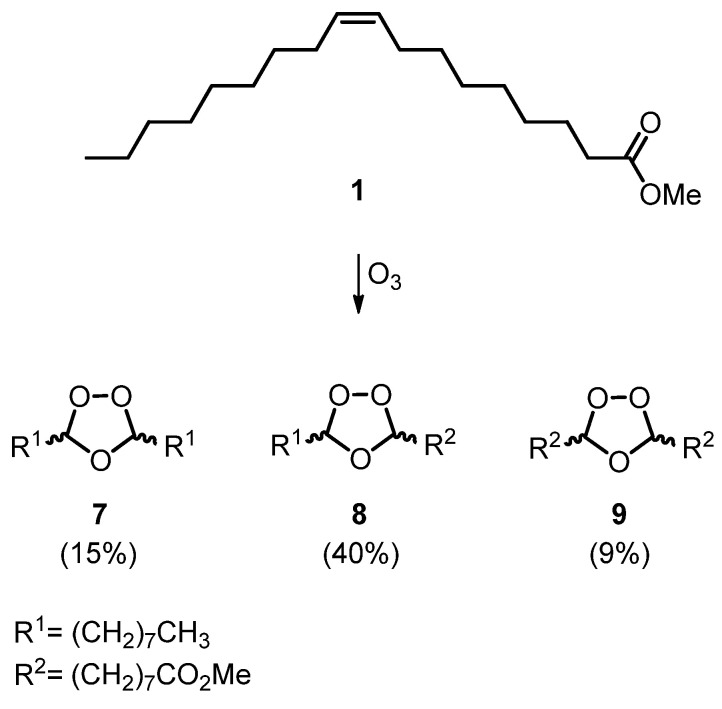
Ozonolysis reaction products of methyl oleate (**1**).

Successively, the ozonolysis of **1** was repeated using an excess of ozone, with the aim to isolate the corresponding ozonides. The NMR spectra of the crude reaction mixture showed only the presence of the same products observed in the reaction described above, except for the starting material, which was completely absent. Silica gel chromatography provided the recovery of compounds **7**, **8** and **9**, each as a mixture of *cis*/*trans*-isomers (Scheme 3), which were recognized through a comparison of their spectral data with those reported in the literature [9].

For mixtures **7** and **8**, the *cis* and *trans*-isomers were separated by preparative TLC chromatography, while for mixture **9**, which appeared solid, the *cis* and *trans*-isomers were separated via recrystallization from DCM/*n*-hexane. 

**Figure 2:**
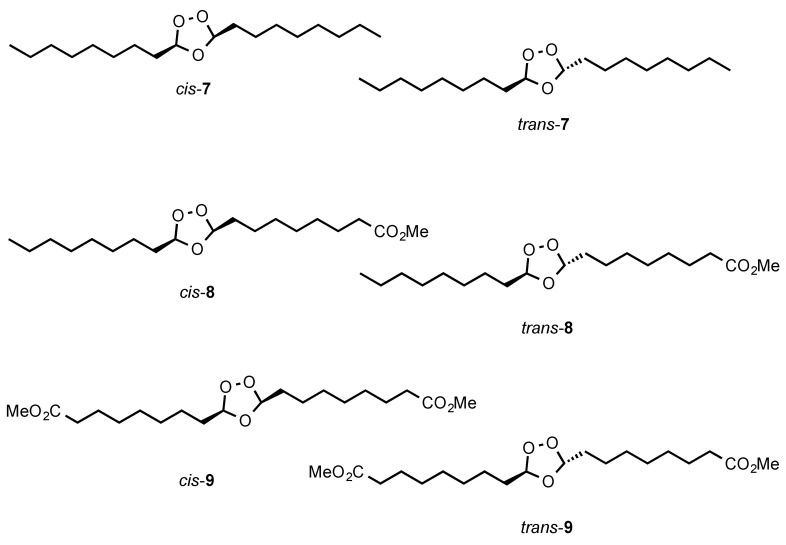
Ozonides isolated from ozonolysis of methyl oleate (**1**).

Figure 2 shows the structures of the six isolated products.

**Figure 3:**
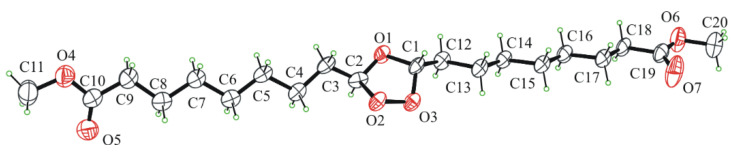
ORTEP view of compound *trans-***9** molecular structure with thermal ellipsoids drawn at 50% probability level.

The structure of all compounds was assigned based on ^1^H and ^13^C NMR data. In addition, the structure of compound *trans*-**9**, which was solid, was confirmed by X-ray crystallography (Figure 3), which also allowed us to confirm that the protons of the peroxidic ring of all *trans*-ozonides in the ^1^H NMR spectra are shielded with respect to those of the corresponding *cis*, as previously reported [5,9].

Furthermore, the assignment of the *cis*/*trans* configurations in **7** and **8** was also consistent with the smaller Rf value of the *cis*-isomers in the normal-phase TLC, in agreement with their larger dipole moment [9].

With the aim to control if the course of the ozonolysis of **1** could be comparable with that of a triglyceride structure, the reaction was repeated using triolein (**2**) chosen as a model, using an excess of ozone, as described above for **1**. After the ozonolysis, the crude reaction mixture of **2** was treated with sodium methoxide using a mixture of methanol/DCM as the solvent. When the reaction was complete, the ^1^H NMR spectra showed the presence of compounds **7**, **8** and **9**. The **7** and **8** molar ratios were similar to that found starting from **1**, while the ozonides **9** were formed in higher yields.

A similar trend was observed in the ozonolysis of +OIL which leads to Ozoile. The methanol trans-esterification led to the main ozonides **7**, **8** and **9** in a similar molar ratio to that observed starting from triolein.

**Scheme 4:**
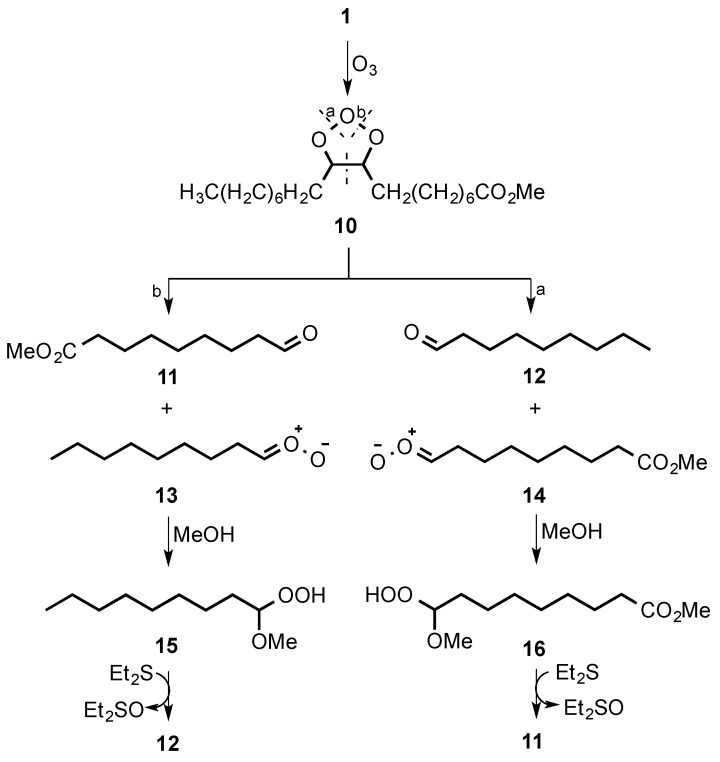
Trapping reaction of carbonyl oxides in the ozonolysis of methyl oleate (**1**).

The well-known reaction mechanism was then investigated by carrying out the ozonolysis of **1** using methanol as the solvent. This solvent works well in the trapping of carbonyl oxides that are formed in the fragmentation of primary ozonides (Scheme 4) [20].

The NMR spectra of an aliquot showed signals in accordance with the two hydroperoxidic derivatives **15** and **16**. Et_2_S addition causes these signals to disappear, while the signal of the CHO group increases. Silica chromatography afforded the hydroperoxides **15**, recognized by comparison with reported spectral data [20], and **16**, which was fully characterized. The comparable yields observed in **15** and **16** denoted the formation of a similar amount of carbonyl oxides **13** and **14**.

**Scheme 5:**
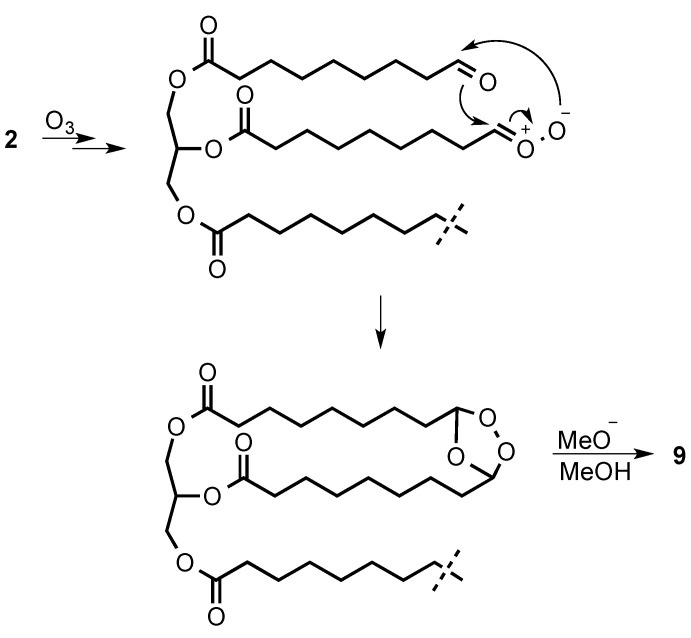
Intramolecular cycloaddition in the ozonolysis of triolein (**2**).

As expected, compounds **8** represent the main products in the ozonolysis of **1**. In fact, **8** can be formed by 1,3-dipolar cycloaddition of both **13** to **11** and **14** to **12**, while **7** and **9** were derived by cycloaddition of **13** to **12** and **14** to **11**, respectively. The higher yields in **9** in the ozonolysis of triolein (**2**), if compared to **1**, could be explained considering their additional formation by an intramolecular 1,3-dipolar cycloaddition, as shown in Scheme 5. This trend was confirmed to occur in Ozoile too. Finally, the *cis*/*trans* ratio was approximately 1:1 for all isolated ozonides **7**–**9**.

### Single Crystal Structure Analysis

The *trans* molecular structure of the 1,2,4-trioxolane ring in compound *trans*-**9** is confirmed by X-ray diffraction data analysis. All bond lengths and angles are in the normal range and are in agreement with similar compounds [21,22].

The puckering analysis revealed that the central trioxolane ring is in the half-chair conformation twisted on O2-O3 (Figure S1 and Table S2). This conformation agrees with the literature findings for a not very encumbered 1,2,4-trioxolane ring [22], while the envelope conformation is found for a less encumbered ring [21]. The whole molecule assumes a linear extended shape as a consequence of the *trans* arrangement of the ring and the all-trans conformation of octanoate groups. Crystal packing is stabilized by weak CH^…^O interactions (Figures S2 and S3).

## 3. Materials and Methods

### 3.1. General Information

Ozone was generated by a CFS-3 Ozonia Ozonat ozone generator. Melting points are uncorrected. Spectra were recorded on a Fourier Transform NMR Varian 500 Unity Inova spectrometer. X-ray analysis was performed on a Bruker-Nonius Kappa CCD diffractometer. Analytical TLC was performed on precoated silica gel plates (Macherey-Nagel, Düren, Germany). Column chromatography was performed on silica gel (0.063–0.2 mm) (Macherey-Nagel). Preparative TLC was performed on precoated silica gel plates (Macherey-Nagel, Düren, Germany) with a 1 mm film thickness.

### 3.2. Treatment of ***1*** and +OIL with ^3^O_2_

In a 0.02 M solution of **1** in dichloromethane (DCM), oxygen was slowly bubbled for 4 h through the solution kept at 0 °C. A second aliquot of the same solution was left at room temperature in contact with air for ten days. Next, TLC and ^1^H NMR analysis of both solutions showed the presence of the only starting material. The same results were found treating +OIL with oxygen following the same procedures. 

### 3.3. Ene-Reaction of ***1***

A 0.02 M solution of **1** in dry DCM was irradiated at −20 °C with a halogen lamp (650 W) in the presence of methylene blue, while dry oxygen was bubbled through the solution. After 5 h, the reaction was stopped, and the solvent was removed under reduced pressure. Silica gel chromatography (*n*-hexane/ether) of the residue afforded the hydroperoxides **3** and **4** in addition to the unreacted **1**, which was the main compound. Reduction by Et_2_S of both hydroperoxides **3** and **4** led to the allylic alcohols **5** and **6**, respectively. All compounds were recognized through comparison with the literature data [17,19].

### 3.4. Ozonolysis of ***1***

An amount of 297 mg (*ca.* 1 mmol) of **1** was transferred into a double-neck reaction flask and an excess of ozone gas at 0 °C was added. The crude mixture was chromatographed on silica gel affording, in addition to the little amount of aldehydes **11** and **12**, the ozonides **7** in 15% yields (*n*-hexane/diethyl ether:19/1 *v*/*v*), **8** in 40% yields (*n*-hexane/diethyl ether:9/1 *v*/*v*) and **9** in 9% yields (*n*-hexane/diethyl ether:8/2 *v*/*v*) as diastereoisomeric mixtures. Next, the *cis*,*trans*-**7** and -**8** mixtures of ozonides were separated by preparative TLC, which led to the corresponding pure diastereoisomers in the *ca* 1:1 molar ratio. The *cis*,*trans*-ozonides **9** were separated into the *ca* 1:1 molar ratio by recrystallization performed by slow evaporation of a DCM/*n*-hexane solution at room temperature, which afforded the *trans*-ozonide **9** as crystals, while the *cis-***9** was recovered from the solution.

*cis*-*Ozonide* **7**: oil; ^1^H NMR (CDCl_3_); δ = 0.88 (t, *J* = 7.02 Hz, 6 H, 2 × Me), 1.25–1.42 (m, 24 H, 12 × CH_2_), 1.66–1.70 (m, 4 H, 2 × CH_2_), 5.19 (t, *J* = 5.10 Hz, 2 H, 2 × CH); ^13^C NMR (CDCl_3_); δ = 14.1 (q), 22.6 (t), 23.9 (t), 29.2 (t), 29.3 (t), 29.4 (t), 31.8 (t), 32.4 (t), 104.3 (d). Anal. Calcd. for C_18_H_36_O_3_: C, 71.95; H, 12.08. Found: C, 71.86; H, 12.10.

*trans*-*Ozonide* **7**: oil; ^1^H NMR (CDCl_3_); δ = 0.85 (t, *J* = 6.74 Hz, 6 H, 2 × Me), 1.23–1.47 (m, 24 H, 12 × CH_2_), 1.68–1.74 (m, 4 H, 2 × CH_2_), 5.14 (t, *J* = 4.80 Hz, 2 H, 2 × CH); ^13^C NMR (CDCl_3_); δ = 14.1 (q), 22.6 (t), 23.8 (t), 29.1 (t), 29.4 (t), 29.5 (t), 30.8 (t), 31.8 (t), 104.3 (d). Anal. Calcd. for C_18_H_36_O_3_: C, 71.95; H, 12.08. Found: C, 71.96; H, 12.12.

*cis*-*Ozonide* **8**: oil; ^1^H NMR (CDCl_3_); δ = 0.88 (t, *J* = 7.00 Hz, 3 H, Me), 1.22–1.44 (m, 22 H, 11 × CH_2_), 1.64–1.70 (m, 4 H, 2 × CH_2_), 2.30 (t, *J* = 7.47 Hz, 2 H, C*H*_2_CO_2_Me) 3.67, (s, 3 H, CO_2_Me), 5.19 (t, *J* = 5.09 Hz, 2 H, 2 × CH); ^13^C NMR (CDCl_3_); δ = 14.1 (q), 22.6 (t), 23.8 (t), 23.9 (t), 24.9 (t), 28.9 (t), 29.0 (t), 29.1 (t), 29.3 (t), 29.4 (t), 31.8 (t), 32.3 (t), 32.4 (t), 34.0 (t), 51.4 (q), 104.2 (d), 104.3 (d), 174.2 (s). Anal. Calcd. for C_19_H_36_O_5_: C, 66.25; H, 10.53. Found: C, 66.28; H, 10.60.

*trans*-*Ozonide* **8**: oil; ^1^H NMR (CDCl_3_); δ = 0.88 (t, *J* = 6.81 Hz, 3 H, Me), 1.23–1.44 (m, 22 H, 11 × CH_2_), 1.67–1.74 (m, 4 H, 2 × CH_2_), 2.30 (t, *J* = 7.49 Hz, 2 H, C*H*_2_CO_2_Me) 3.66 (s, 3 H, CO_2_Me), 5.14 (t, *J* = 4.79 Hz, 2 H, 2 × CH); ^13^C NMR (CDCl_3_); δ = 14.0 (q), 22.6 (t), 23.7 (t), 23.8 (t), 24.9 (t), 28.9 (t), 29.0 (t), 29.1 (t), 29.2 (t), 29.4 (t), 29.5 (t), 30.7 (t), 30.8 (t), 31.8 (t), 34.1 (t), 51.4 (q), 104.2 (d), 104.3 (d), 174.2 (s). Anal. Calcd. for C_19_H_36_O_5_: C, 66.25; H, 10.53. Found: C, 66.30; H, 10.72.

*cis*-*Ozonide* **9**: oil; ^1^H NMR (CDCl_3_); δ = 1.23–1.42 (m, 20 H, 10 × CH_2_), 1.57–1.71 (m, 4 H, 2 × CH_2_), 2.29 (t, *J* = 7.53 Hz, 4 H, 2 × C*H*_2_CO_2_Me), 3.66 (s, 3 H, CO_2_Me), 5.17 (t, *J* = 5.04 Hz, 2 H, 2 × CH); ^13^C NMR (CDCl_3_); δ = 23.8 (t), 24.9 (t), 28.9 (t), 29.0 (t), 29.1 (t), 32.3 (t), 34.1 (t), 51.5 (q), 104.1 (d), 104.2 (d), 174.3 (s). Anal. Calcd. for C_20_H_36_O_7_: C, 61.83; H, 9.34. Found: C, 62.01; H, 9.40.

*trans*-*Ozonide* **9**: m.p. 55–56 °C; ^1^H NMR (CDCl_3_); δ = 1.28–1.45 (m, 20 H, 10 × CH_2_), 1.67–1.73 (m, 4 H, 2 × CH_2_), 2.29 (t, *J* = 7.55 Hz, 4 H, 2 × C*H*_2_CO_2_Me), 3.66 (s, 3 H, CO_2_Me), 5.13 (t, *J* = 4.72 Hz, 2 H, 2 × CH); ^13^C NMR (CDCl_3_); δ = 24.6 (t), 25.8 (t), 29.9 (t), 30.0 (t), 30.2 (t), 31.7 (t), 35.0 (t), 52.4 (q), 105.2 (d), 175.2 (s). Anal. Calcd. for C_20_H_36_O_7_: C, 61.83; H, 9.34. Found: C, 61.92; H, 9.42.

### 3.5. Ozonolysis of ***1*** in MeOH

A solution of 297 mg (*ca*. 1 mmol) of **1** in dry MeOH (0.25 M) was transferred into a double-neck reaction flask and an excess of ozone gas at 0 °C was added. The ^1^H NMR (CDCl_3_) of an aliquot showed, in addition to the aldehydes **11** and **12**, the presence of the hydroperoxides **15** [20] and **16** in the *ca* 1:1 molar ratio, which were isolated by a quick silica gel chromatography. Hydroperoxide **16** was characterized by ^1^H- and ^13^C NMR spectroscopy.

*Methoxy-hydroperoxide* **16**: oil; ^1^H NMR (CDCl_3_); δ = 1.24–1.42 (m, 6 H, 3 × CH_2_), 1.53–1.73 (m, 6 H, 3 × CH_2_), 2.29 (t, *J =* 7.50 Hz, 2 H, C*H*_2_CO_2_Me), 3.49 (s, 3 H, OMe), 3.65 (s, 3 H, CO_2_Me), 4.72 (t, *J =* 5.83 Hz, 1 H, CH), 8.56 (br s, 1 H, OOH); ^13^C NMR (CDCl_3_); δ = 24.5 (t), 24.8 (t), 28.8 (t), 28.9 (t), 29.0 (t), 31.2 (t), 34.0 (t), 51.5 (q), 55.8 (q), 108.7 (d), 174.4 (s). Anal. Calcd. for C_11_H_22_O_5_: C, 56.39; H, 9.47. Found: C, 56.42; H, 9.52.

### 3.6. Ozonolysis of Triolein (***2***) Followed by Trans-Esterification

An amount of 443 mg (*ca.* 0.5 mmol) of **2** was ozonized as reported above for **1** (par. *3.4*). Next, 162 mg of sodium methoxide (1:2 equiv.) was added to a DCM/MeOH (1/3 *v*/*v*, ca. 0.05 M) solution of the crude reaction mixture, which was kept under stirring at rt. After 60 min, the reaction was completed (TLC). The solvent was removed and the residue was extracted by Et_2_O (3 × 20 mL). The organic layer was dried by MgSO_4_ and filtered. The solvent was removed and the residue was chromatographed as reported above for **1**, affording the ozonides **7** (14% yields), **8** (42% yields) and **9** (20% yields).

### 3.7. Ozonolysis of +OIL (Synthesis of Ozoile) Followed by Trans-Esterification

An amount of 500 mg of Ozoile (stable Ozonides) was obtained through a patented process (EP 3900821-Erbagil s.r.l.) through the reaction of an excess of ozone with organic extra virgin olive oil (+OIL by Erbagil Estate). The crude mixture was dissolved in dry DCM (4 mL) and dry methanol was added (12 mL). Thus, a large excess of sodium methoxide was added to this solution (200 mg) and the resulting mixture was kept under stirring at rt. After 60 min, the reaction was completed (TLC). The solvent was removed, and the residue was dissolved and extracted by Et_2_O (3 × 30 mL). Then, the organic layer was dried by MgSO_4_ and filtered. The solvent was removed and the residue was chromatographed as reported above for **1**, mainly affording the ozonides **7** (15% yields), **8** (38% yields), and **9** (20% yields) with a similar molar ratio observed starting from triolein (**2**).

### 3.8. X-Ray Crystallography of trans-***9***

Single crystals of *trans*-ozonide **9** were obtained as colorless, thick platelets through a slow evaporation of a DCM/*n*-hexane solution at room temperature. One suitable crystal was selected and mounted on a Bruker-Nonius KappaCCD four-circle diffractometer in flowing N_2_ at 173 K. The SADABS program [23] was used for absorption correction. The structure was solved by direct methods with the SIR97 program [24] and using the SHELXL-2019/2 program [25] with the aid of the software package WinGX [26]. Anisotropic parameters were used for non-H atoms. All H atoms were generated geometrically and refined according to the riding model. The crystals were of low quality and showed poor diffracting power. Very weak diffraction intensities from high angles were observed, even if collected at low temperatures. Nevertheless, the low-quality data that explains the rather high R values observed the structure refined to convergence, and the results agree with other analytical data.

Crystal data and structure refinement details are reported in Tables S1–S3. The figures were generated using ORTEP-3 [26] and Mercury 4.0 [27]. Crystallographic data were deposited in CCDC under the deposit number 2372929 for *trans*-ozonide **9**.

## 4. Conclusions

In conclusion, this study reports a methodology directed to isolate and characterize the ozonides formed in the ozonolysis of **1** as a strategy to identify the main stable ozonides in Ozoile. For the first time, the *cis*/*trans* mixture of each derivative has been separated, allowing for the isolation of each diastereoisomer, which has been fully characterized. In addition to the NMR spectral data collected for all derivatives, an X-ray analysis has been run on the solid derivative **9** truly providing the assignment of the *trans*-configuration. A similar trend of ozonide distribution has been found in the ozonolysis of triolein **2**, followed by methanol trans-esterification. Triolein **2** represents the main component of biological extra virgin olive oil (+OIL) which leads to Ozoile by ozonolysis. This is provided to identify the main stable ozonides present in Ozoile, whose matrix is highly complex to investigate. Moreover, products deriving from reactions with singlet oxygen, as well as triplet oxygen, were not found, not even in traces. The isolated pure diastereoisomers will be tested in suitable biological systems to better understand the biological activities of Ozoile.

## Data Availability

Data is contained within the article or the Supplementary Data File.

## Supplementary Materials

The following supporting information can be downloaded at: https://www.mdpi.com/article/10.3390/molecules30030507/s1, Table S1. Crystal data and structure refinement details for ozonide *trans*-**9**. Crystallographic data for the structure have also been deposited with the Cambridge Crystallographic Data Centre as supplementary publication number CCDC2372929; Table S2. Selected bond lengths and angles with standard deviations for ozonide *trans*-**9**; Table S3. Hydrogen bonds for ozonide *trans*-**9**; Figure S1 Detail of ozonide 9 molecule showing the trans-trioxane ring in the half-chair conformation (twisting on O2 -O3, ball and stick style). The plane containing O1/C1/C2 atoms is drawn in blue with distances of O2 and O3 from this plane in green; Figure S2 Crystal packing of ozonide *trans*-**9** viewed along a axis showing layers of molecules piled up in the c axis direction; Figure S3 Partial packing of ozonide *trans*-**9** viewed along a axis direction showing the herringbone arrangement of molecules. ^1^H and ^13^C NMR spectra for all new compounds. X-ray crystallographic data for *trans*-**9**. Supplementary data associated with this article can be found in the online version, at CCDC 2372929 contains the supplementary crystallographic data for this paper. These data can be obtained free of charge from The Cambridge Crystallographic Data Centre via “www.ccdc.cam.ac.uk/data_request/cif”.
