# Supplementary material for: Characterization of Pure Ozonides from Ozonolysis of Oleic Acid Methyl Ester; Use of a Protocol for the Analysis of the Corresponding Stable Ozonides from Triolein and Organic Extra Virgin Olive Oil (+OIL®)
Source: Molecules. 2025 Jan 23;30(3):507. doi: 10.3390/molecules30030507 (PMC11819892; doi:10.3390/molecules30030507)
Supplement: Supplementary file 1 [file molecules-30-00507-s001.zip › molecules-3307335-supplementary.pdf]

Characterization of Stable Ozonides (Ozoile®) Produced through a Patented Process of Ozonolysis of Organic Extra Virgin Olive Oil (+OIL®)

Serena Vella [2], Marina DellaGreca [1], Angela Tuzi [1] and Flavio Cermola [1,*]

[1] Dipartimento di Scienze Chimiche, Università di Napoli Federico II, Complesso Universitario di M. Sant'Angelo, Via Cintia, 80126 Napoli, Italy; dellagre@unina.it; angela.tuzi@unina.it.

[2] Erbagil s.r.l., Via L. Settembrini, 13, 82037 Telese Terme, Italy; s.vella@erbagil.com

* Correspondence: cermola@unina.it

Supplementary Materials

Table of Contents

^{1}H NMR ($CDCl_3$) of *cis*-ozonide **7** S1

^{13}C NMR ($CDCl_3$) of *cis*-ozonide **7** S2

^{1}H NMR ($CDCl_3$) of *trans*-ozonide **7** S3

^{13}C NMR ($CDCl_3$) of *trans*-ozonide **7** S4

^{1}H NMR ($CDCl_3$) of *cis*-ozonide **8** S5

^{13}C NMR ($CDCl_3$) of *cis*-ozonide **8** S6

^{1}H NMR ($CDCl_3$) of *trans*-ozonide **8** S7

^{13}C NMR ($CDCl_3$) of *trans*-ozonide **8** S8

^{1}H NMR ($CDCl_3$) of *cis*-ozonide **9** S9

^{13}C NMR ($CDCl_3$) of *cis*-ozonide **9** S10

^{1}H NMR ($CDCl_3$) of *trans*-ozonide **9** S11

^{13}C NMR ($CDCl_3$) of *trans*-ozonide **9** S12

^{1}H NMR ($CDCl_3$) of hydroperoxide **16** S13

^{13}C NMR ($CDCl_3$) of hydroperoxide **16** S14

X-Ray diffraction analysis of ozonide *trans*-**9** S15

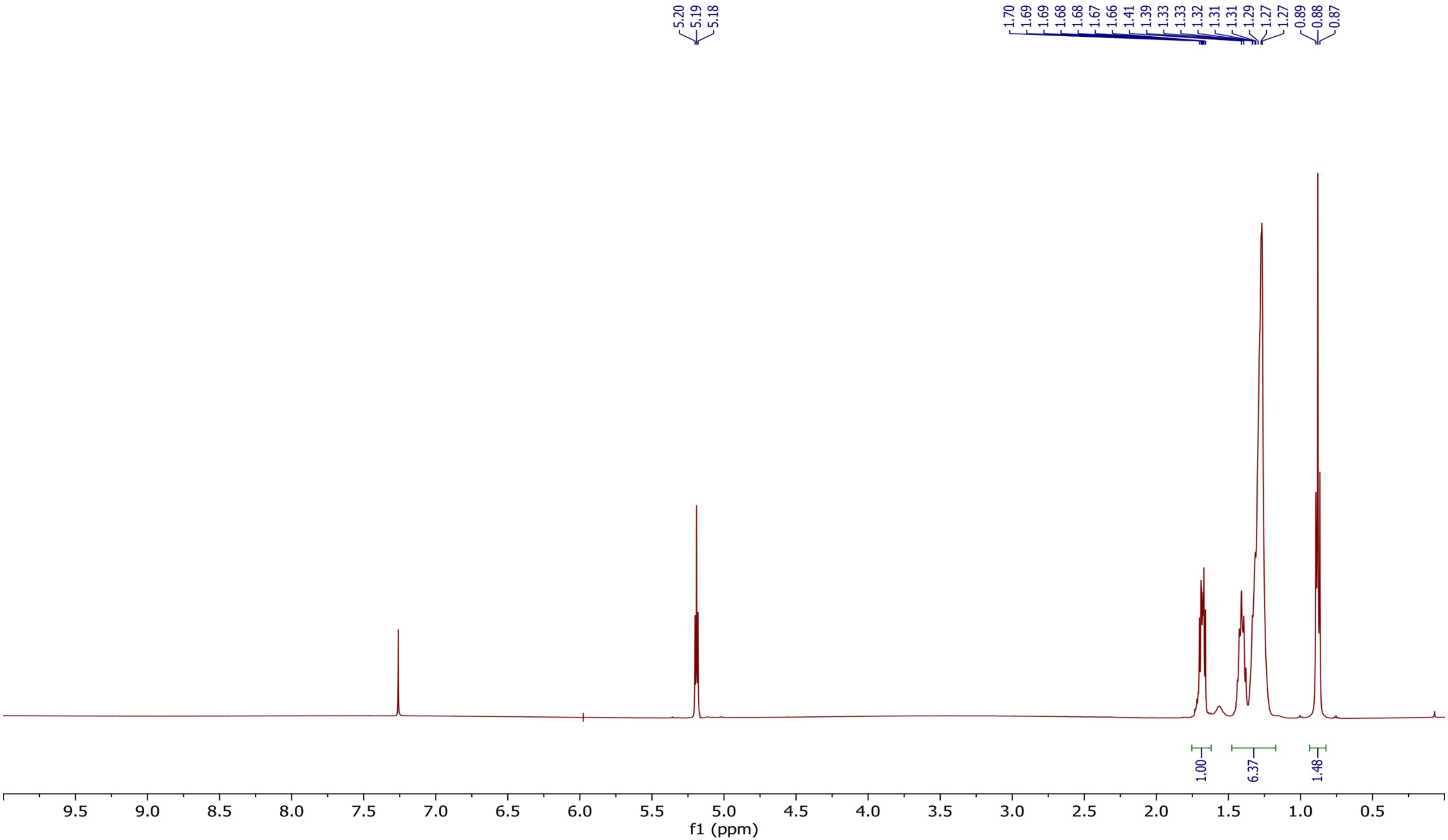

^{1}H NMR ($CDCl_3$) of *cis*-ozonide **7**

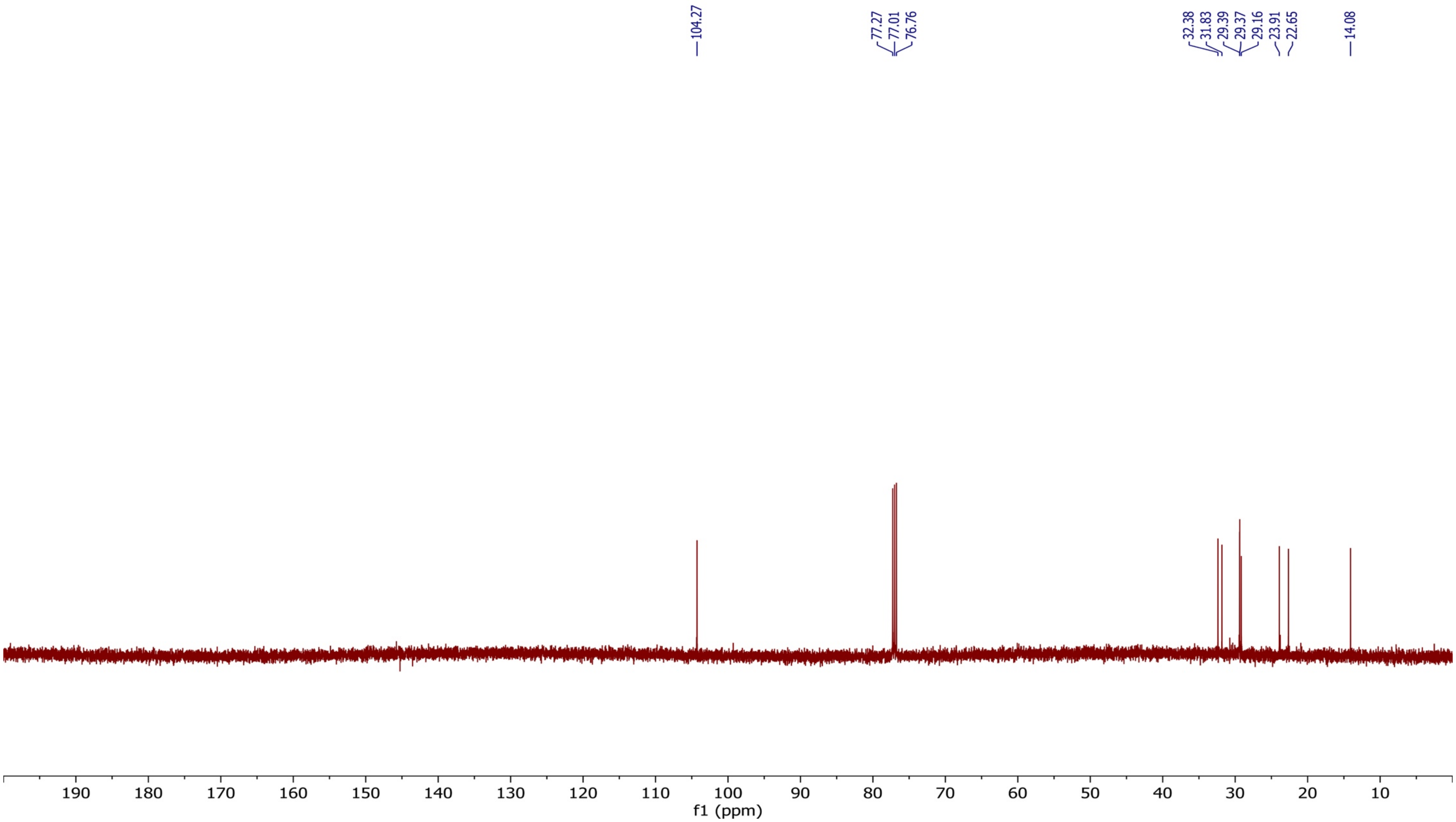

^{13}C NMR ($CDCl_3$) of *cis*-ozonide **7**

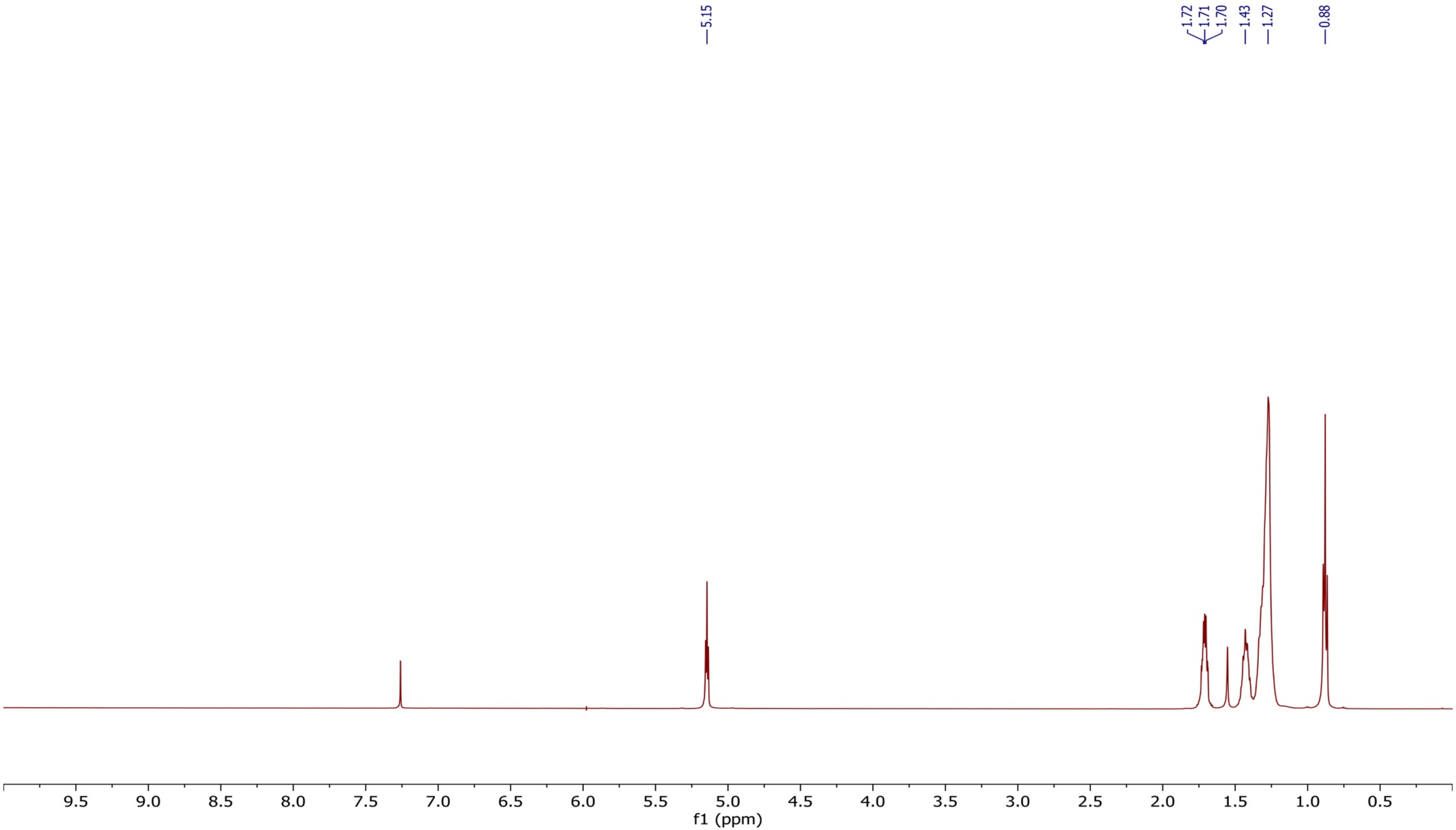

^{1}H NMR ($CDCl_3$) of *trans*-ozonide **7**

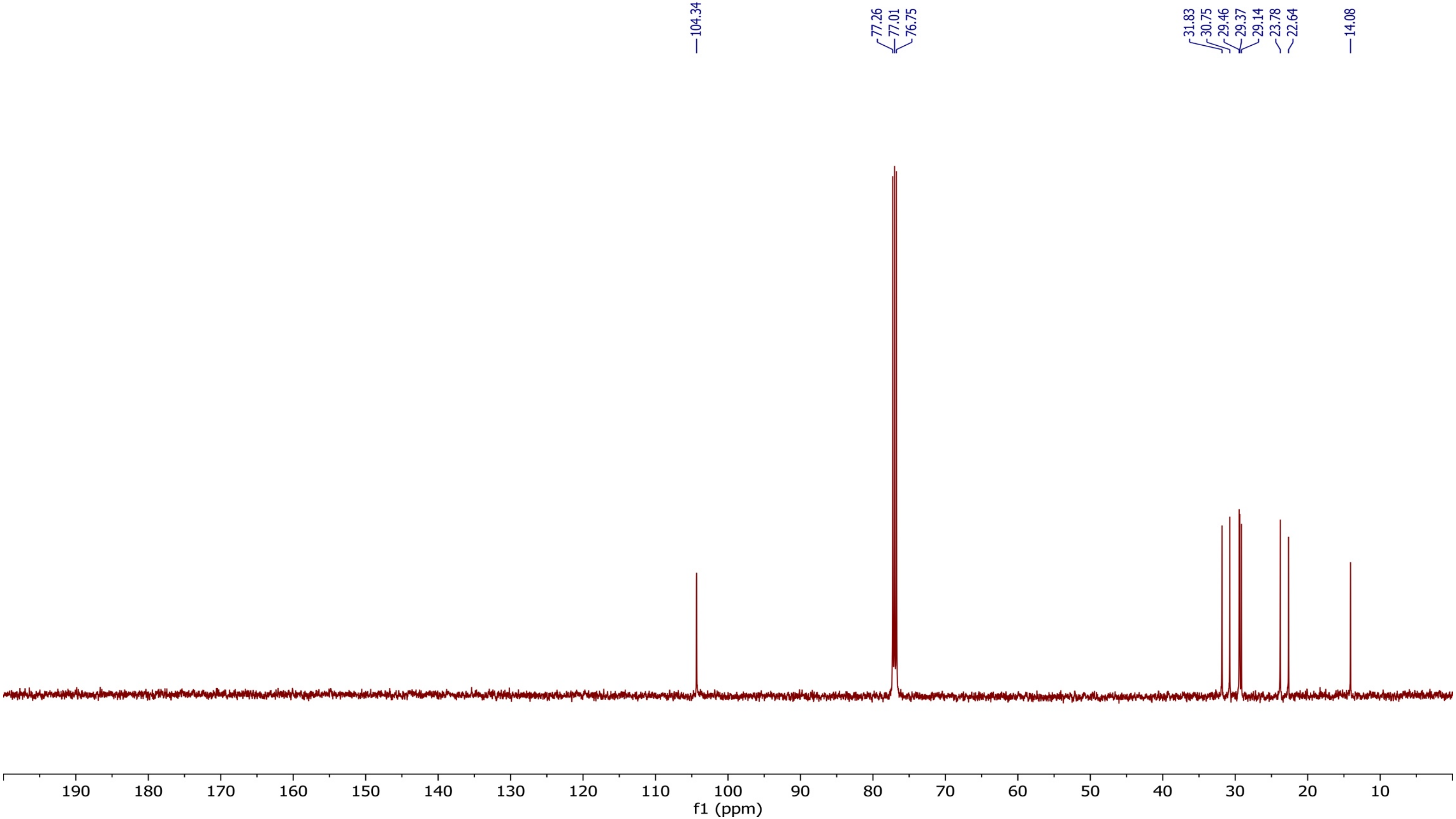

^{13}C NMR ($CDCl_3$) of *trans*-ozonide **7**

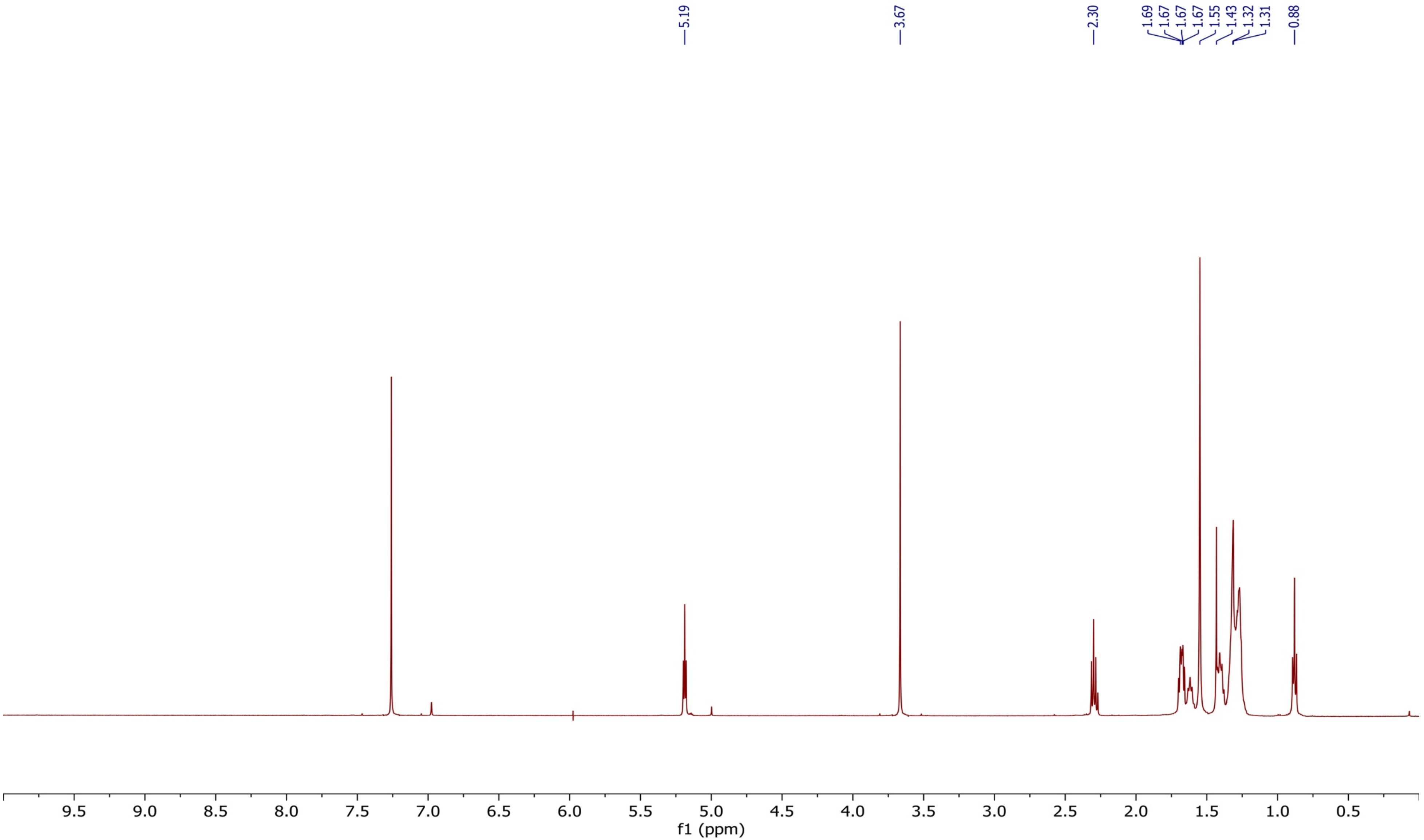

^{1}H NMR ($CDCl_3$) of *cis*-ozonide **8**

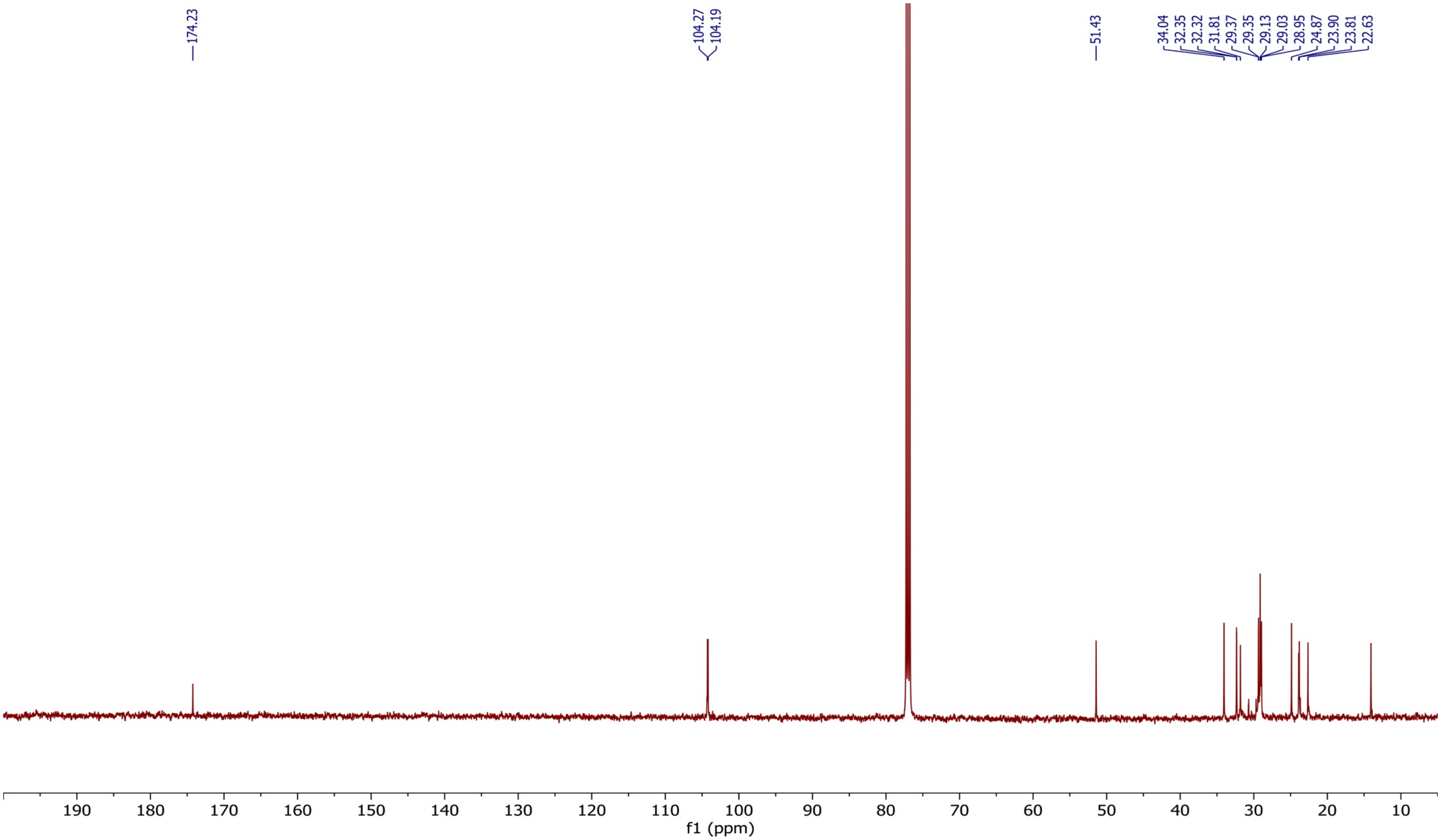

^{13}C NMR ($CDCl_3$) of *cis*-ozonide **8**

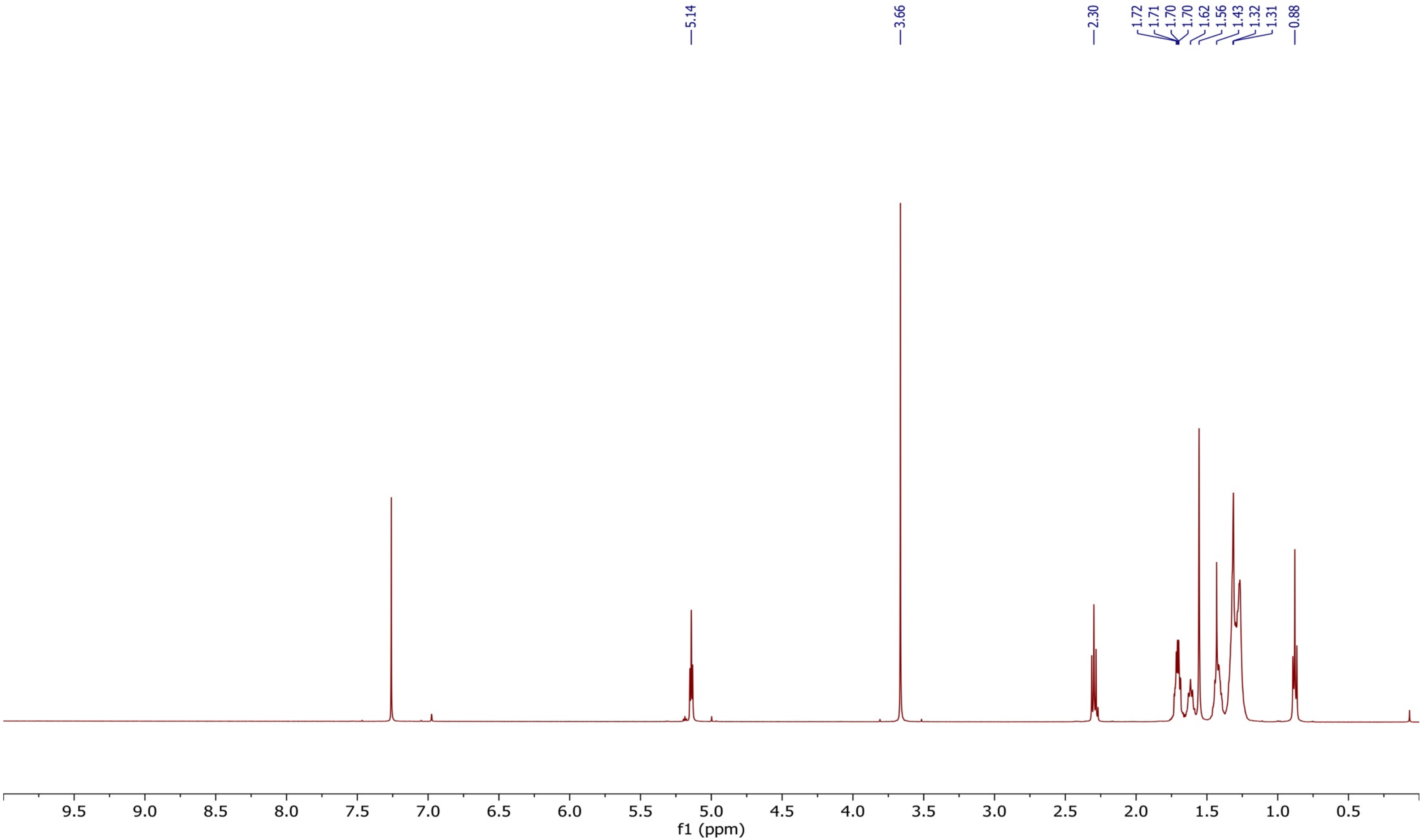

^{1}H NMR ($CDCl_3$) of *trans*-ozonide **8**

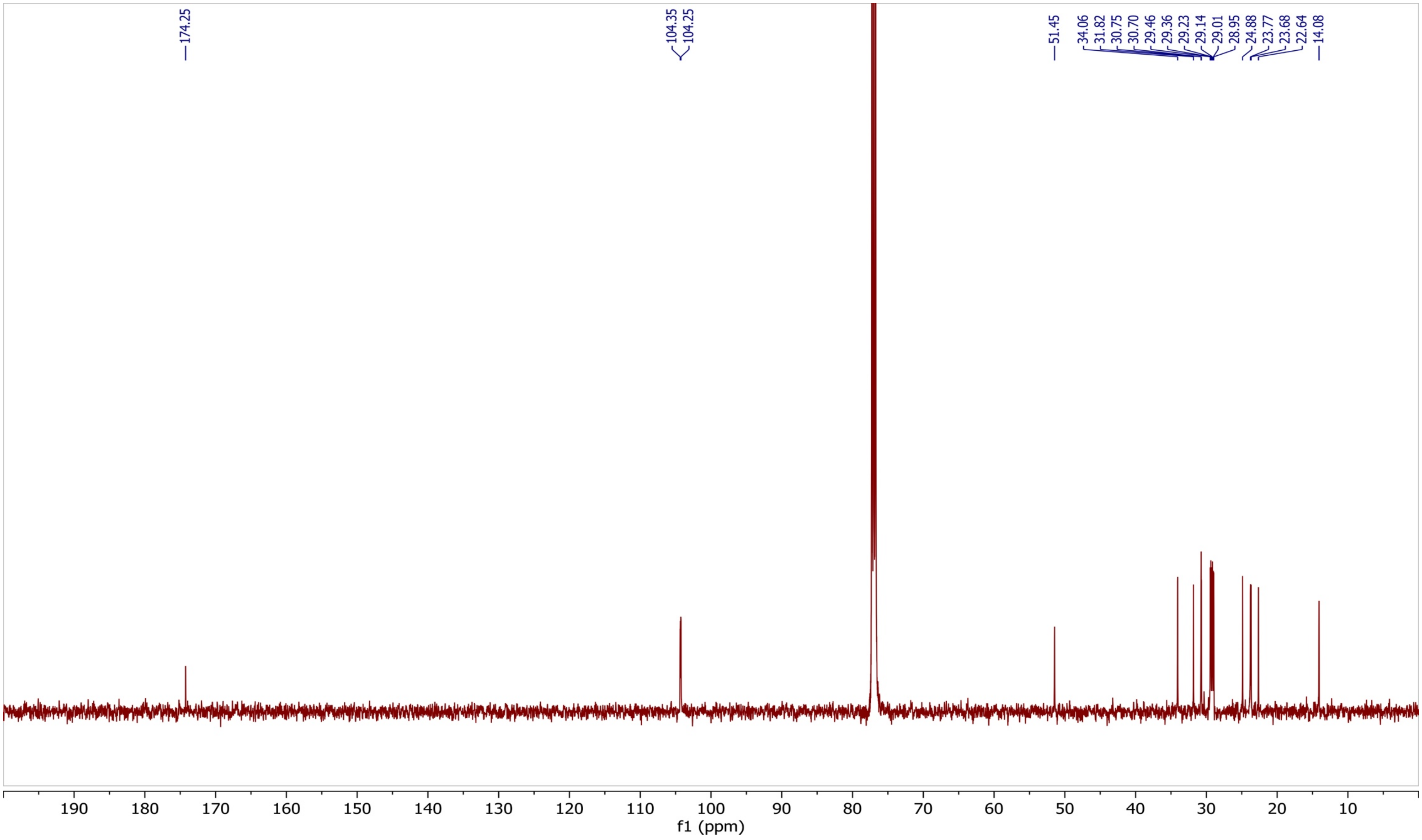

^{13}C NMR ($CDCl_3$) of *trans*-ozonide **8**

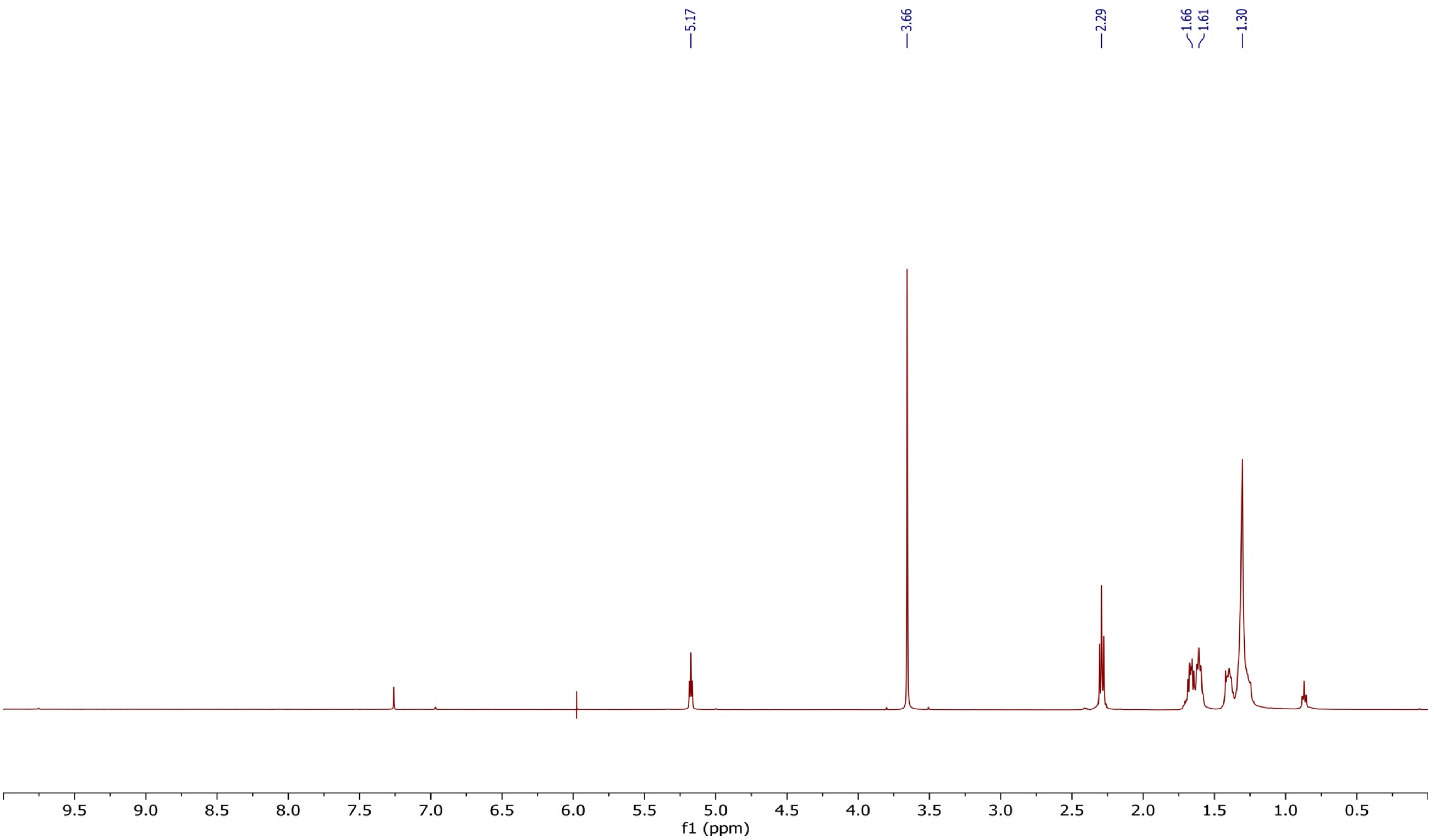

^{1}H NMR ($CDCl_3$) of *cis*-ozonide **9**

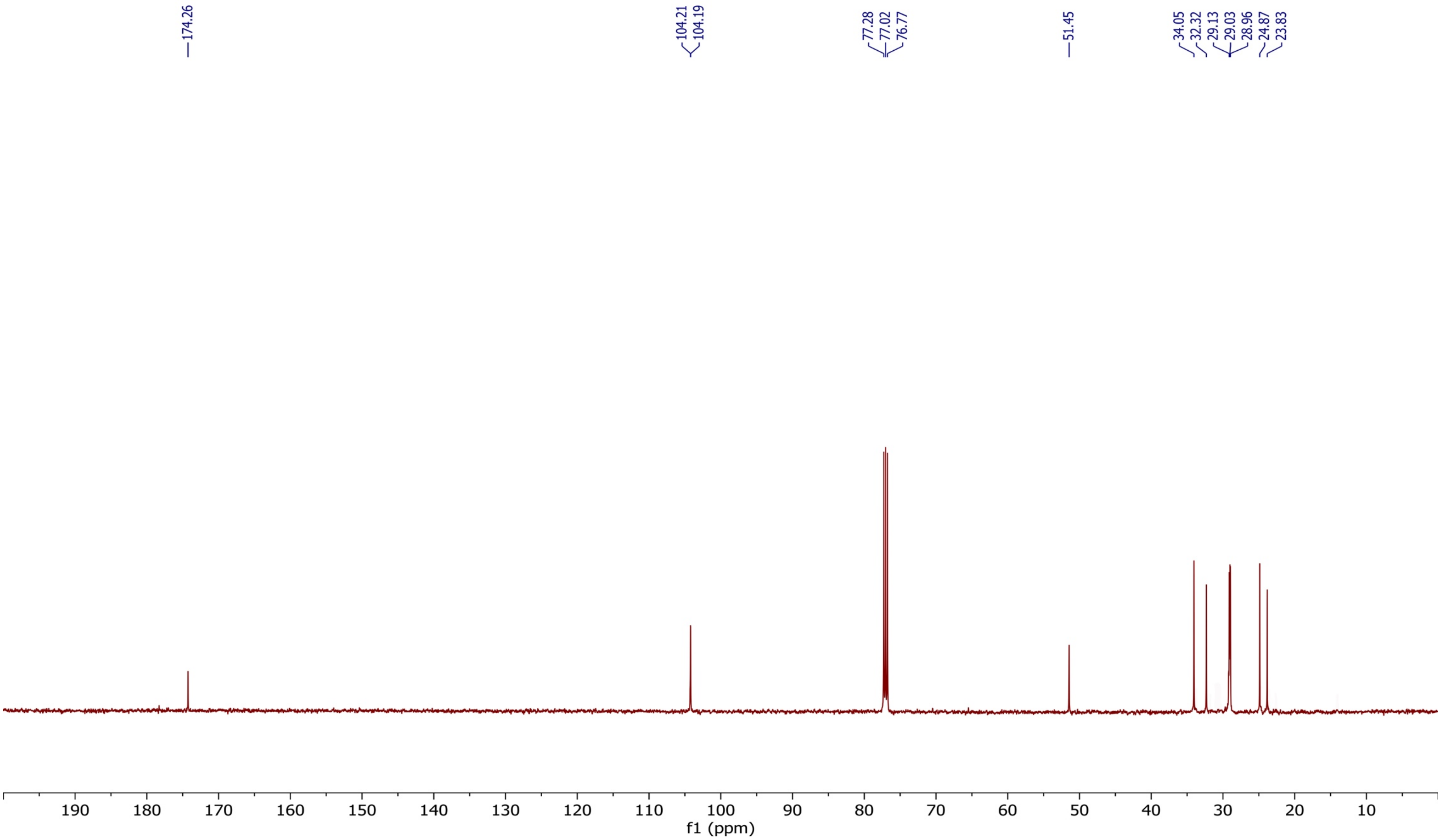

^{13}C NMR ($CDCl_3$) of *cis*-ozonide **9**

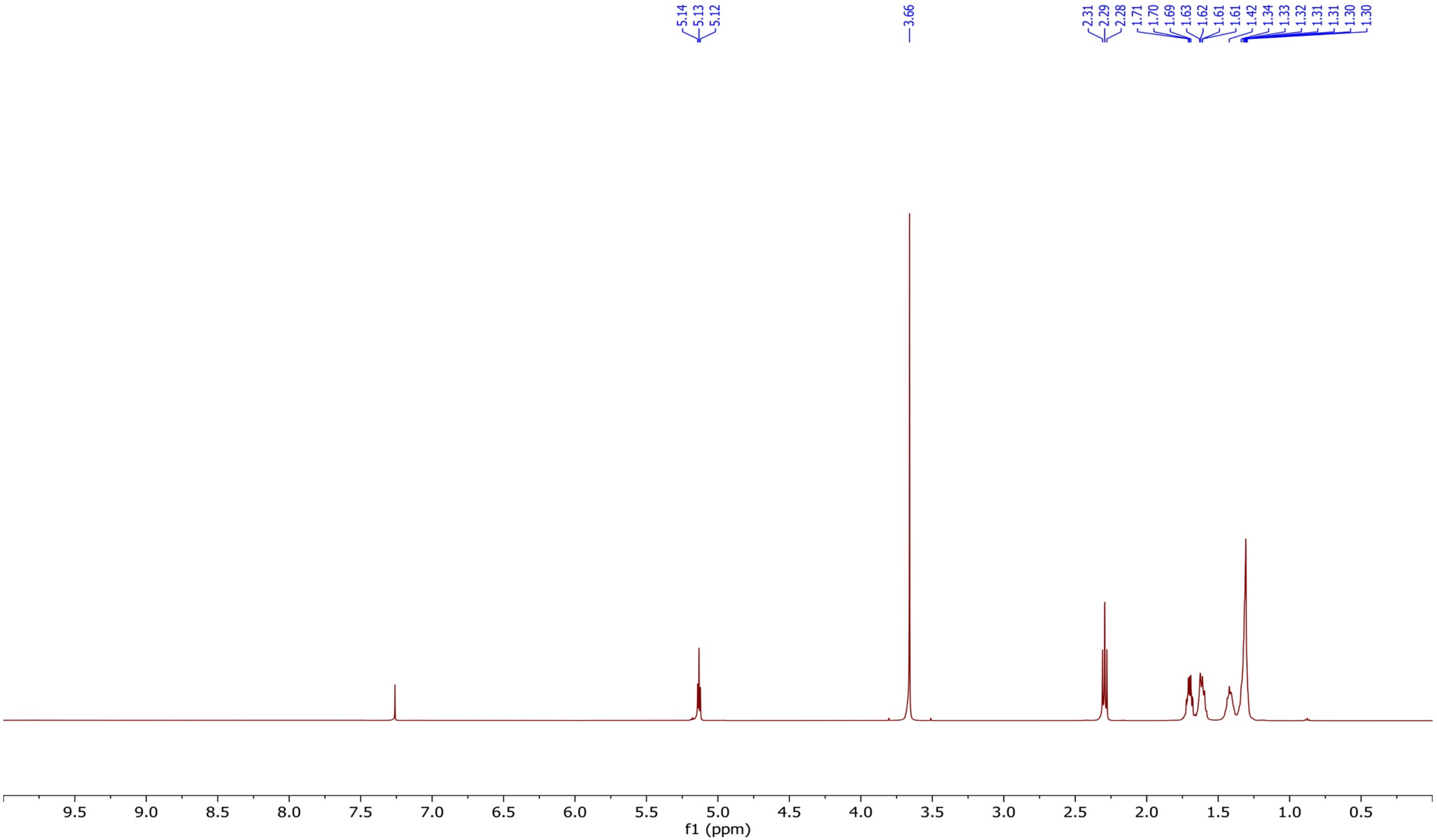

^{1}H NMR ($CDCl_3$) of *trans*-ozonide **9**

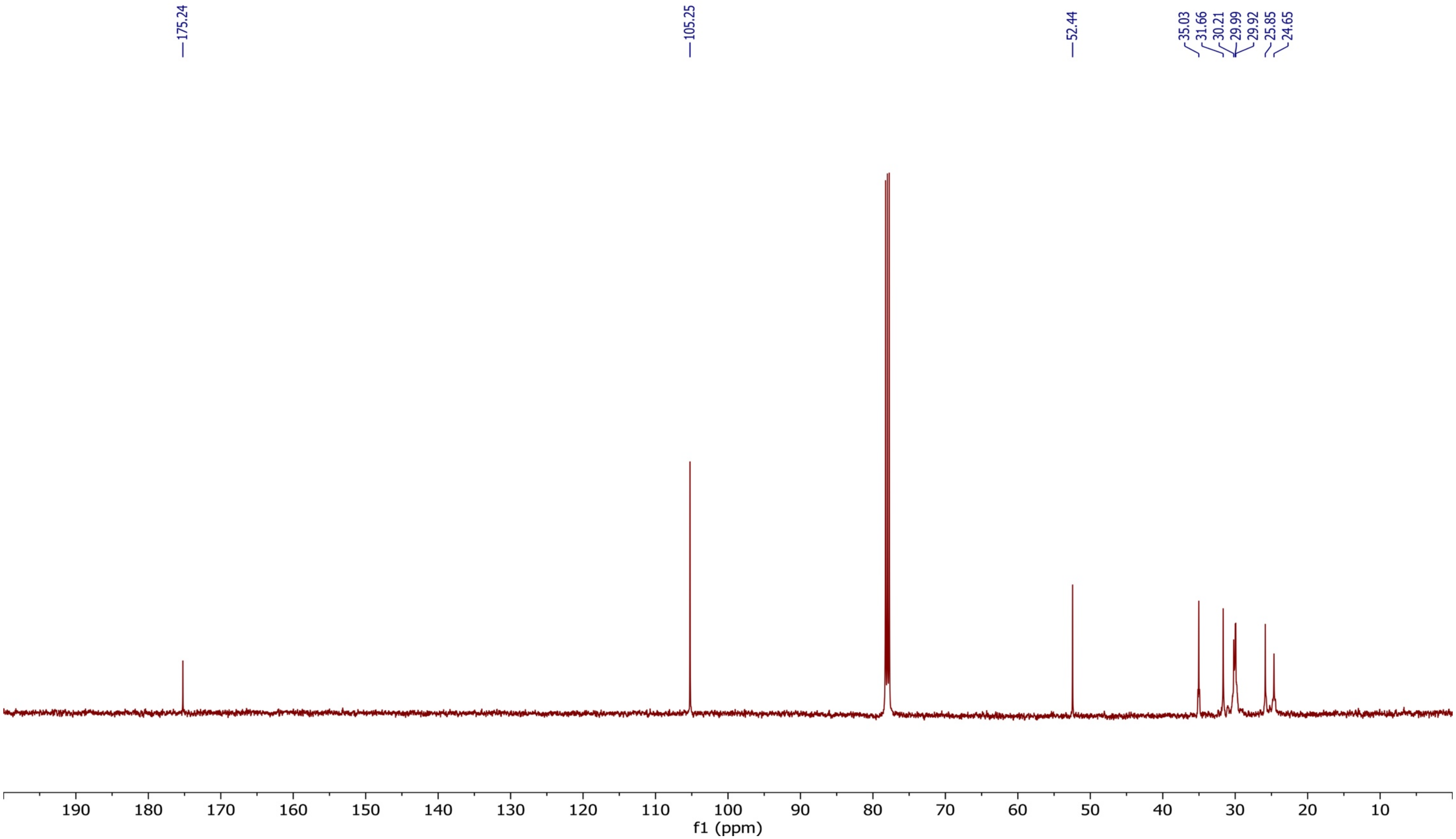

^{13}C NMR ($CDCl_3$) of *trans*-ozonide **9**

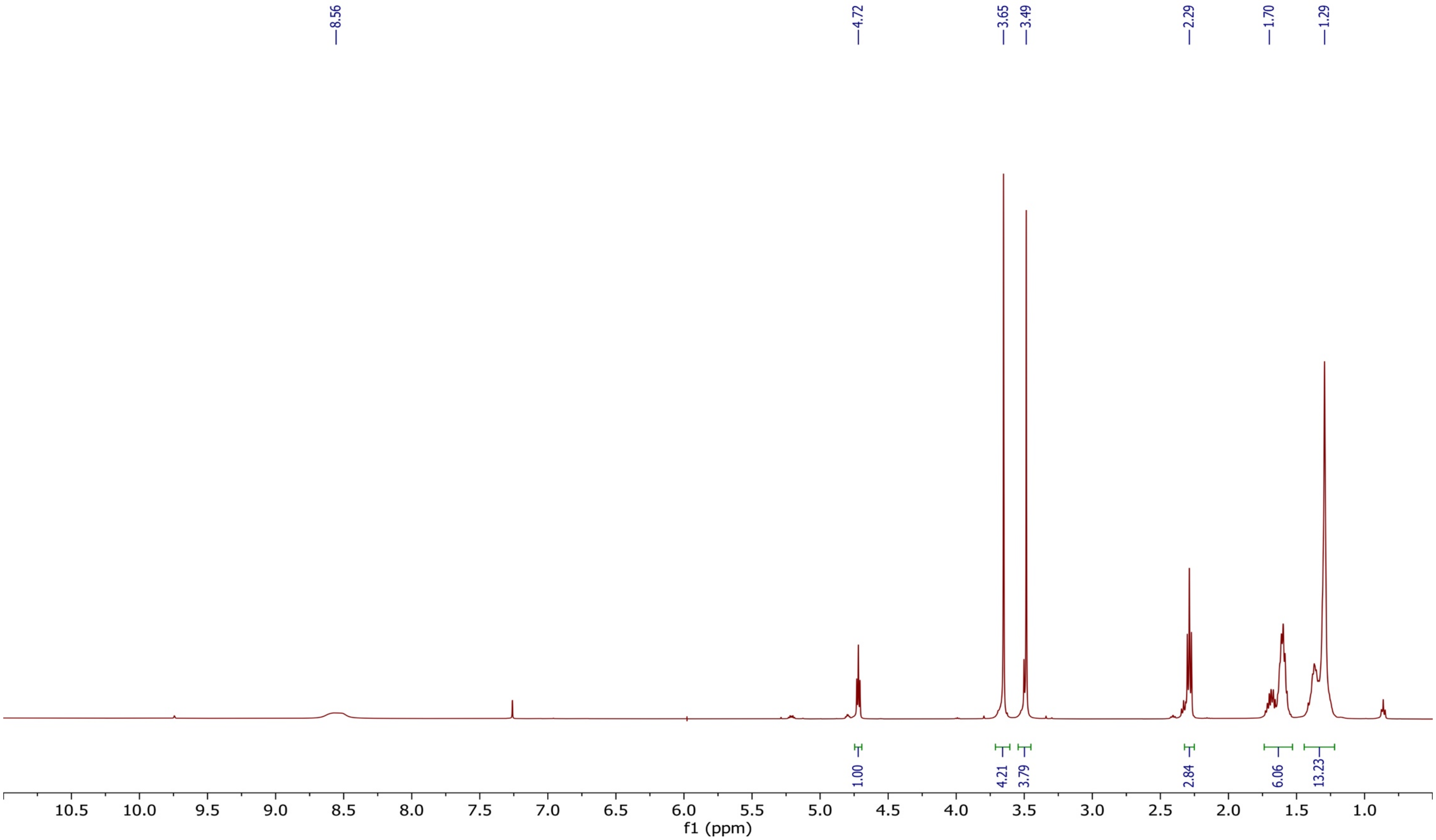

^{1}H NMR ($CDCl_3$) of hydroperoxide **16**

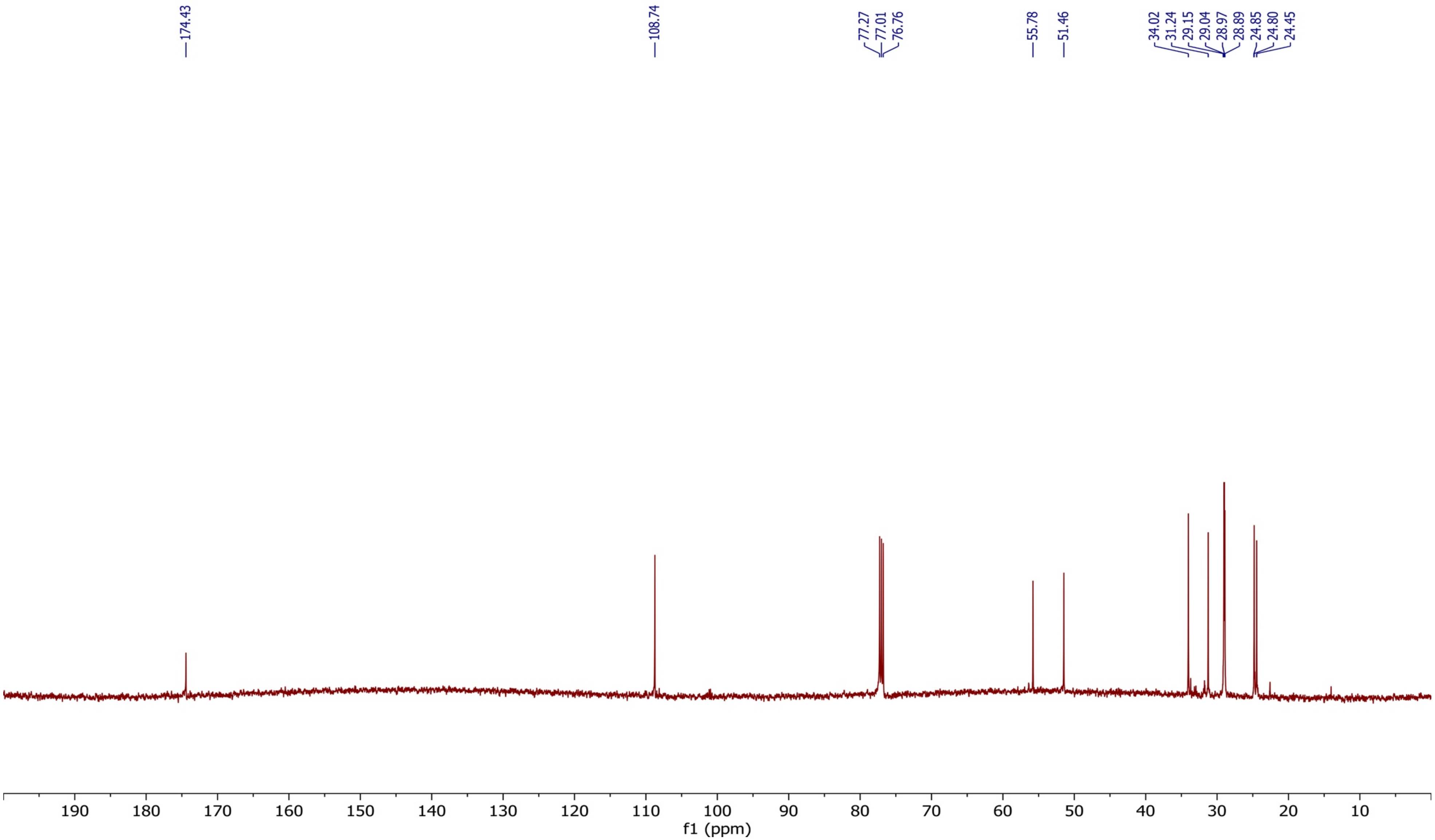

^{13}C NMR ($CDCl_3$) of hydroperoxide **16**

Table S1. Crystal data and structure refinement details for ozonide *trans*-**9**. Crystallographic data for the structure have also been deposited with the Cambridge Crystallographic Data Centre as supplementary publication number CCDC2372929.

Empirical formula	$C_{20} H_{36} O_7$
Formula weight	388.49
Temperature	2173(2) K
Wavelength	0.71073 Å
Crystal system, space group	Monoclinic, P 21/c
Unit cell dimensions	a = 7.086(4) Å α = 90°
	b = 41.879(8) Å β = 98.33(3)°
	c = 7.337(4) Å γ = 90°
Volume	2154.3(17) $Å^3$
Z, Calculated density	4, 1.198 Mg/m^3
Absorption coefficient	0.089 mm^{-1}
F(000)	848
Crystal size	0.270 x 0.150 x 0.020 mm
Theta range for data collection	3.064 to 24.997°
Limiting indices	-8<=h<=7, -49<=k<=49, -8<=l<=8
Reflections collected / unique	11721 / 3707 [R(int) = 0.1017]
Completeness to theta=25.186	97.8%
Refinement method	Full-matrix least-squares on F^2
Data / restraints / parameters	3707 / 0 / 247
Goodness-of-fit on F^2	1.043
Final R indices [I>2sigma(I)]	R1 = 0.1138, wR2 = 0.2636
R indices (all data)	R1 = 0.1855, wR2 = 0.3052
Extinction coefficient	n/a
Largest diff. peak and hole	0.551 and -0.405 e $Å^{-3}$

Table S2. Selected bond lengths and angles with standard deviations for ozonide *trans*-**9**.

C(1)-O(3)	1.408(6)
C(1)-O(1)	1.442(6)
C(2)-O(1)	1.410(6)
C(2)-O(2)	1.435(6)
O(2)-O(3)	1.476(5)
C(2)-O(1)-C(1)	106.6(4)
C(2)-O(2)-O(3)	99.5(3)
O(3)-C(1)-O(1)	103.9(4)
O(1)-C(2)-O(2)	104.8(4)
O(1)-C(2)-O(2)-O(3)	38.8(4)
O(1)-C(1)-O(3)-O(2)	41.2(4)
O(2)-C(2)-O(1)-C(1)	-14.2(5)
O(3)-C(1)-O(1)-C(2)	-17.6(5)

Table S3. Hydrogen bonds for ozonide *trans*-**9**.

D-H...A	d(D-H)	d(H...A)	d(D...A)	<(DHA)
C(9)-H(9B)...O(7)#1	0.99	2.48	3.279(8)	138.0
C(20)-H(20C)...O(5)#2	0.98	2.65	3.190(8)	114.7

Symmetry transformations used to generate equivalent atoms: #1 = (-x+1,-y,-z+1) ; #2 = (-x+1,y-1/2,-z+1/2)

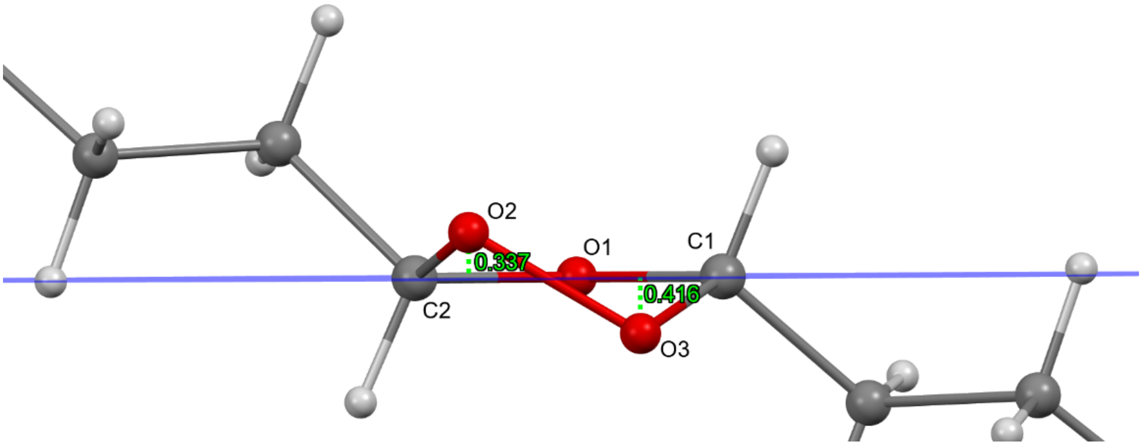

Figure S1 Detail of ozonide **9** molecule showing the *trans*-trioxane ring in the half-chair conformation (twisting on O2 -O3, ball and stick style). The plane containing O1/C1/C2 atoms is drawn in blue with distances of O2 and O3 from this plane in green.

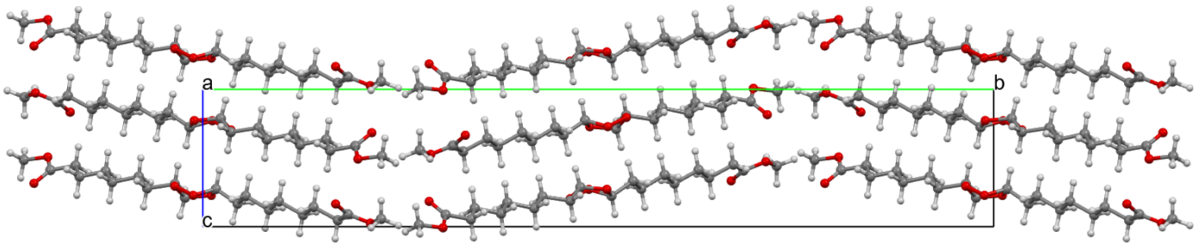

Figure S2 Crystal packing of ozonide *trans*-**9** viewed along **a** axis showing layers of molecules piled up in the **c** axis direction.

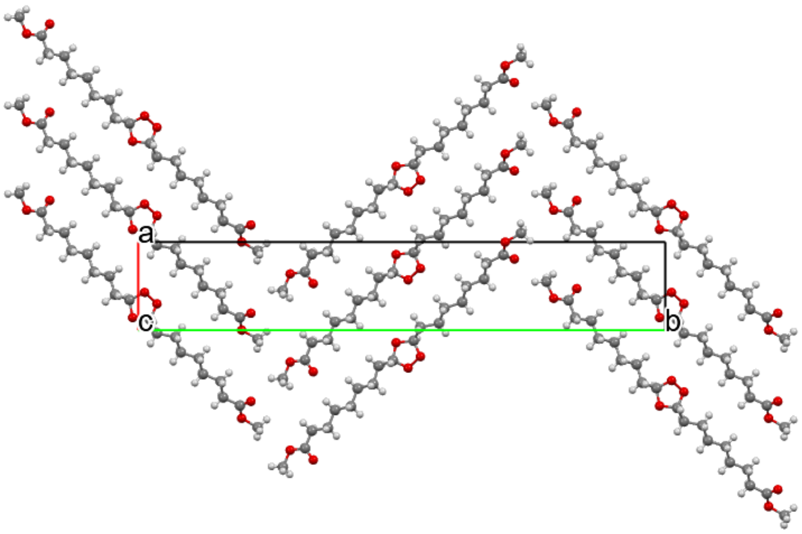

Figure S3 Partial packing of ozonide *trans*-**9** viewed along **a** axis direction showing the herringbone arrangement of molecules.
